# Build a better bootstrap and the RAWR shall beat a random path to your door: phylogenetic support estimation revisited

**DOI:** 10.1093/bioinformatics/btab263

**Published:** 2021-07-12

**Authors:** Wei Wang, Ahmad Hejasebazzi, Julia Zheng, Kevin J Liu

**Affiliations:** Department of Computer Science and Engineering, Michigan State University, East Lansing, MI 48824, USA; Department of Computer Science and Engineering, Michigan State University, East Lansing, MI 48824, USA; Department of Computer Science and Engineering, Michigan State University, East Lansing, MI 48824, USA; Ecology, Evolution, and Behavior Program, Michigan State University, East Lansing, MI 48824, USA; Department of Computer Science and Engineering, Michigan State University, East Lansing, MI 48824, USA; Ecology, Evolution, and Behavior Program, Michigan State University, East Lansing, MI 48824, USA; Genetics and Genome Sciences Program, Michigan State University, East Lansing, MI 48824, USA

## Abstract

**Motivation:**

The standard bootstrap method is used throughout science and engineering to perform general-purpose non-parametric resampling and re-estimation. Among the most widely cited and widely used such applications is the phylogenetic bootstrap method, which Felsenstein proposed in 1985 as a means to place statistical confidence intervals on an estimated phylogeny (or estimate ‘phylogenetic support’). A key simplifying assumption of the bootstrap method is that input data are independent and identically distributed (i.i.d.). However, the i.i.d. assumption is an over-simplification for biomolecular sequence analysis, as Felsenstein noted.

**Results:**

In this study, we introduce a new sequence-aware non-parametric resampling technique, which we refer to as RAWR (‘RAndom Walk Resampling’). RAWR consists of random walks that synthesize and extend the standard bootstrap method and the ‘mirrored inputs’ idea of Landan and Graur. We apply RAWR to the task of phylogenetic support estimation. RAWR’s performance is compared to the state-of-the-art using synthetic and empirical data that span a range of dataset sizes and evolutionary divergence. We show that RAWR support estimates offer comparable or typically superior type I and type II error compared to phylogenetic bootstrap support. We also conduct a re-analysis of large-scale genomic sequence data from a recent study of Darwin’s finches. Our findings clarify phylogenetic uncertainty in a charismatic clade that serves as an important model for complex adaptive evolution.

**Availability and implementation:**

Data and software are publicly available under open-source software and open data licenses at: https://gitlab.msu.edu/liulab/RAWR-study-datasets-and-scripts.

## 1 Introduction

In 1985, Felsenstein proposed the application of standard bootstrap resampling (Efron, 1979) to place confidence intervals on an estimated phylogeny (Felsenstein, 1985). Given an input multiple sequence alignment (MSA), the approach first generates bootstrap replicates by sampling input MSA columns uniformly at random with replacement. Then, phylogenetic re-estimation is performed on each bootstrap replicate. Finally, bootstrap support for each edge of an annotation phylogeny (i.e. the phylogeny estimated on the original input MSA) is calculated as the fraction of re-estimated phylogenies that also display that edge.

Bootstrap support estimation has become a de facto standard for assessing reproducibility in modern phylogenetics and phylogenomics, and Felsenstein’s seminal 1985 paper has become the 41st most cited in all of science, according to the survey of Van Noorden *et al.* (2014). Alternatives include other non-parametric resampling methods such as the jackknife (Tukey, 1958) and parametric resampling (Felsenstein, 2004; Warnow, 2017). Examples of the latter include MSA-specific confidence measures [e.g. GUIDANCE1 (Landan and Graur, 2008; Penn *et al.*, 2010), GUIDANCE2 (Sela *et al.*, 2015), PSAR (Kim and Ma, 2011), T-COFFEE (Notredame *et al.*, 2000), wpSBOOT (Chang *et al.*, 2019) and Divvier (Ali *et al.*, 2019)], parametric and/or special-purpose MSA resampling or filtering techniques applied to the problem of phylogenetic support estimation [e.g. TCS (Chang *et al.*, 2014), the unistrap (Chatzou *et al.*, 2018), Gblocks (Talavera and Castresana, 2007), Trimal (Capella-Gutiérrez *et al.*, 2009), and the method of Rajan (2013)], and other alignment-oblivious phylogenetic support estimation methods [e.g. aLRT (Anisimova and Gascuel, 2006) and Bayesian posterior probability-based alternatives (Yang and Rannala, 1997)]. Finally, alternatives have also been proposed for the last step of phylogenetic support calculation. For example, (Lemoine *et al.*, 2018) introduced the transfer bootstrap expectation (TBE) method, which pairs bootstrap resampling of MSAs and phylogenetic tree re-estimation with an alternative support calculation. The calculation replaces the traditional binary test for bipartition presence/absence with a finer-grained bipartition transfer distance (Day, 1981). All of these alternative methods are less widely used than the bootstrap. Furthermore, parametric resampling/filtering methods require the assumption that data are generated from a particular parametric model, and special-purpose resampling/filtering methods do not readily generalize beyond a specific application. Our study primarily focuses on non-parametric techniques for phylogenetic support estimation for these reasons.

But the bootstrap also makes a key simplifying assumption. Felsenstein concluded his landmark paper with a forward-thinking cautionary note:

‘A more serious difficulty is the lack of independence of the evolutionary processes in different characters. … For the purposes of this paper, we will ignore these correlations and assume that they cause no problems; in practice, they pose the most serious challenge to the use of bootstrap methods.’

Crucially, a variety of biological factors violate the i.i.d. assumption, including evolutionary processes such as sequence insertions/deletions, genetic recombination and many others. Phylogenomic studies now often utilize partitioned or multi-locus phylogenetic analyses which can incrementally alleviate this issue by applying bootstrapping within partitions/loci, assuming that *a priori*-defined and/or estimated partition/locus boundaries reasonably delineate sequence dependence ‘breakpoints’. But phylogenomic approaches still commonly assume i.i.d. site evolution within a locus, and introduce yet another layer where the same simplifying assumption is applied—i.e. the assumption that gene trees for different loci evolved i.i.d. within a species phylogeny (Maddison, 1997).

We and our co-authors created the SERES (or ‘SEquential RESampling’) method to further relax this simplifying assumption (Wang *et al.*, 2018). SERES generalizes the bootstrap and the ‘mirrored inputs’ idea of Landan and Graur (2007) into a random walk on either unaligned or aligned biomolecular sequence inputs. A critical property of the random walk procedure is ‘neighbor preservation’: neighboring bases that appear in a resampled sequence are also guaranteed to be neighbors in the corresponding untransformed sequence in the original input. SERES resampling of unaligned sequences requires synchronization points in the form of anchor regions, similar to the use of barriers in asynchronous computing. To date, the utility of SERES resampling and re-estimation has been demonstrated on two applications—placing confidence intervals on estimated multiple sequence alignments (Wang *et al.*, 2018) and temporal model inference and learning (Wang *et al.*, 2019; Wuyun *et al.*, 2019). Each application addressed biomolecular sequence dependence due to a specific biological process or factor—sequence insertion and deletion processes in the case of the former, and genetic recombination in the case of the latter. But there are many other important applications in which sequence dependence arises due to these and other factors, and more progress is needed to fully address the challenge that Felsenstein noted.

## 2 Approach

In this study, we return to where we started: Felsenstein’s landmark 1985 contribution—phylogenetic support estimation and the phylogenetic bootstrap method. We now briefly summarize the contributions of our study. Our approach to the problem of phylogenetic support estimation makes use of a simpler resampling strategy which we refer to as RAWR (or ‘RAndom Walk Resampling’). The relative simplicity of RAWR compared to SERES, our previous method for semi-parametric resampling, is the first contribution of our study. RAWR does not require a parametric method for anchor estimation, whereas SERES does, adding complexity in the form of method parameters such as anchor width, count and sequence conservation criterion used for selecting anchors. Second, our approach focuses on sequence-aware non-parametric resampling and re-estimation using unaligned sequence data, which differs from existing studies and widely used state-of-the-art methods. Third, RAWR phylogenetic support estimates offer similar or typically better accuracy compared to the bootstrap method and other leading methods.

## 3 Materials and methods

The computational problem addressed in our study is defined as follows. The problem inputs consist of an MSA *A* that was estimated using an MSA method *f* and a phylogenetic tree T=(V,E) that was estimated using a phylogenetic inference method *g* applied to *A*. The problem output consists of confidence interval estimates (or support estimates) ϵ(e)∈[0,1] for every non-leaf edge e∈E.

### 3.1 Methods under study

**Fig. 1.**
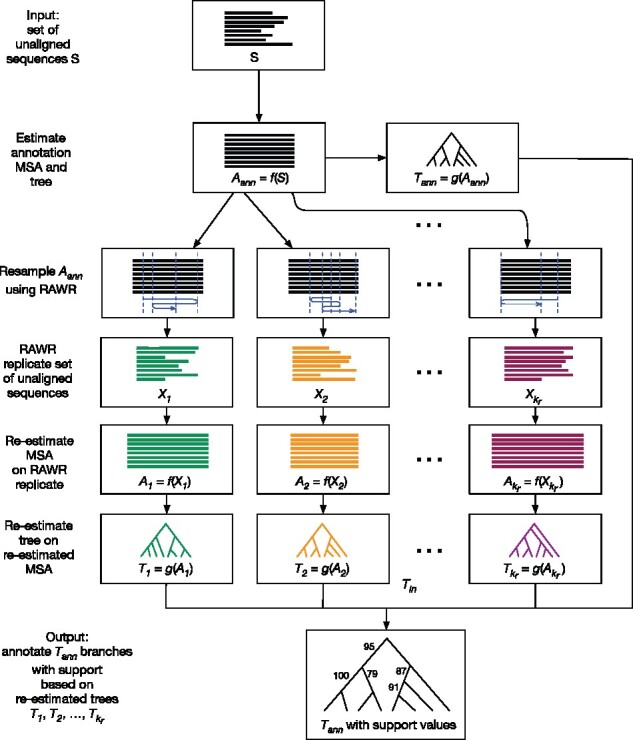
Flowchart of RAWR support estimation steps. RAWR phylogenetic support estimation takes a set of unaligned sequences as input (row 1). To begin, an annotation multiple sequence alignment (MSA) and phylogenetic tree are estimated on the unaligned sequences (row 2). The annotation MSA is resampled to obtain a RAWR replicate set of unaligned sequences (rows 3 and 4), and then MSA and phylogenetic tree re-estimation is performed on the RAWR replicate (rows 5 and 6); resampling and re-estimation is repeated. (See Fig. 2 for a concrete example of steps corresponding to rows 3 through 6.) Phylogenetic support for each non-trivial branch of the annotation tree is calculated based on the proportion of re-estimated trees that also display the branch (row 7)

**Fig. 2.**
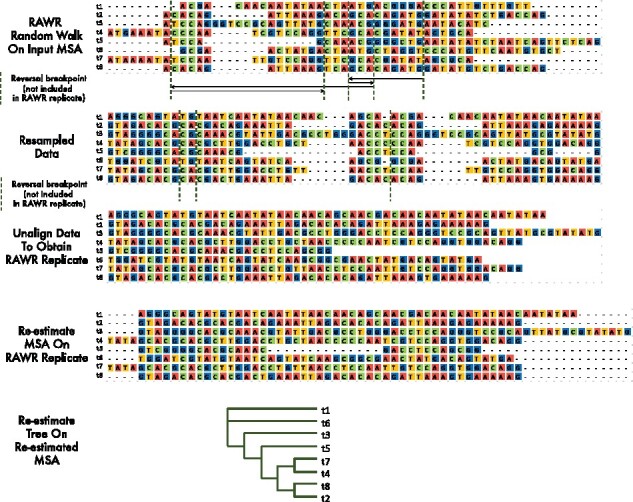
Illustrated example of RAWR resampling and re-estimation. RAWR support estimation begins with a random walk on the annotation MSA (row 1). MSA columns are resampled during the random walk (row 2), and the resampled data is then unaligned to produce a RAWR replicate dataset (row 3). Finally, re-estimation is performed using a two-phase procedure: an MSA is re-estimated on the RAWR replicate dataset (row 4), and a phylogenetic tree is re-estimated using the re-estimated MSA as input (row 5). Reversal breakpoints during the random walk are shown with dashed lines in rows 1 and 2 for illustration purposes, but are not provided as part of the RAWR replicate dataset and are unobserved during re-estimation

**RAWR**. The RAWR method estimates phylogenetic support by resampling the input sequence data and then performing re-estimation on resampled replicate datasets. Pseudocode is provided in Algorithm 1. A flowchart and illustrated example are shown in Figures 1 and 2, respectively.

**Fig. 3.**
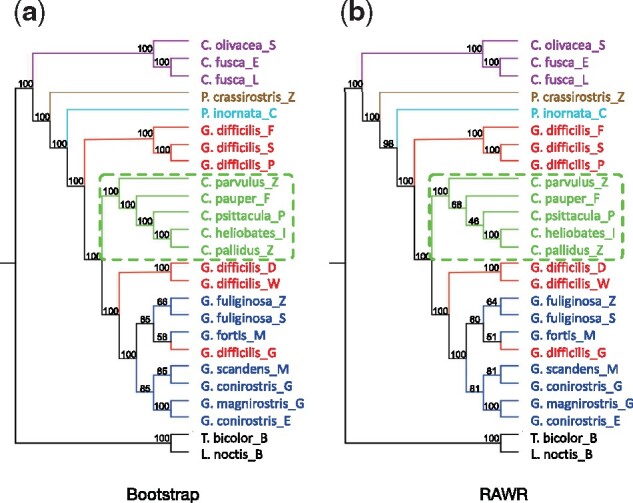
Comparison of (**a**) bootstrap and (**b**) RAWR phylogenetic support estimates on the Darwin’s finch phylogeny. The species phylogeny shown was estimated using a concatenated and partitioned MLE analysis of Lamichhaney *et al.* (2015)’s genomic sequence dataset. Taxon names and colors are reproduced from Figure 1 in Lamichhaney *et al.* (2015): tree finch species are shown in green, sharp-beaked ground finches are shown in red, other ground finches are shown in blue, Cocos finch is shown in light blue, vegetarian finch is shown in brown, warbler finch is shown in purple and outgroup species are shown in black. Genus names are abbreviated as follows: *Geospiza* is ‘G.’, *Certhidea* is purple ‘C.’, *Camarhynchus* is green ‘C.’, *Platyspiza* is ‘P.’, *Tiaris* is ‘T.’ and *Loxigilla* is ‘L.’. Taxon names indicate species name followed by an underscore and a symbol indicating Galápagos island of origin: Darwin is ‘D’, Wolf is ‘W’, Pinta is ‘P’, Santiago is ‘S’, Daphne is ‘M’, Santa Cruz is ‘Z’, Fernandina is red ‘F’, Isabela is ‘I’, San Cristóbal is ‘L’, Floreana is green ‘F’ and España is ‘E’. Also, Cocos Island is abbreviated ‘C’. The tree finch clade (highlighted with a green dashed rectangle) contains the only bipartition that appears in our MLE analysis but not the estimated species tree reported by Lamichhaney *et al.* (2015); the same clade also contains the largest disagreement between bootstrap and RAWR support estimates. In all other clades, estimated bipartitions in our MLE analysis match those reported in Lamichhaney *et al.* (2015), and bootstrap and RAWR support estimate differences are smaller. Tree visualizations were produced using Dendroscope (Huson and Scornavacca, 2012), to which group colors were manually added.

To begin, a random walk is conducted on the input MSA *A* to generate a resampled replicate dataset. The random walk procedure begins by selecting an initial starting position and walk direction at random. Sites are sampled as the walk proceeds along the current walk direction, and the walk reverses direction with certainty when encountering the first and last site and with probability *γ* elsewhere. The random walk procedure concludes once the convergence criterion is satisfied. In this study, we utilized a sequence length criterion which requires that the number of sampled sites be equal to the length of *A*; other criteria are possible (e.g. statistical criteria based on the random walk procedure). Once resampling has converged to yield a resampled set of aligned sequences, the resampled sequences are unaligned (i.e. indels are omitted) to obtain a set of unaligned sequences that may have differing length. (The Supplementary Material also includes additional experiments involving an alternative random walk resampling procedure.)

Each random walk produces a resampled replicate set of unaligned sequences, and the resampling procedure is repeated to obtain a set of resampled replicates. MSA and tree re-estimation is then performed on the resampled replicates. Phylogenetic support for a branch in the original estimated tree is calculated to be the fraction of re-estimated trees that also display the branch. Our performance study utilized reversal probability $\gamma \in \{1\times 10^{-3},1\times 10^{-2},2\times 10^{-2},5\times 10^{-2},1\times 10^{-1},2\times 10^{-1},3\times 10^{-1}\}$. The default choice for *γ* in our study was $1\times 10^{-1}$ unless otherwise noted. RAWR was used to generate 100 resampled replicates for each input dataset.

**Methods for MSA and phylogenetic estimation/re-estimation**. We focused on two-phase methods for phylogenetic inference on unaligned DNA sequences. This class of methods is by far the most prevalent in systematic studies. The first phase of a two-phase method estimates a multiple sequence alignment on an input set of unaligned sequences, and the second phase uses the previous phase’s estimated MSA to estimate a phylogenetic tree. Our performance study included MAFFT (Katoh and Standley, 2013), a popular suite of MSA algorithms which has been shown to be among the most accurate methods for both MSA estimation (Liu *et al.*, 2009, 2012) and, when used in combination with leading MLE phylogenetic inference methods, phylogenetic inference from unaligned sequences (Liu *et al.*, 2009, 2012). We ran version 7.222 of MAFFT software with default settings. (The Supplementary Methods section in the Supplementary Material document lists software commands that were used in our study.) Summary statistics for the estimated MSAs are shown in Table 1. Maximum likelihood phylogenies under the GTR + Γ model (Rodriguez *et al.*, 1990; Wakeley, 1993; Yang, 1993) were inferred on estimated MSAs using RAxML (Stamatakis, 2014). We used version 8.2.11 of the RAxML software. For brevity, RAxML(*x*) denotes a two-phase analysis consisting of running method *x* to first estimate an MSA on an input set of unaligned sequences, followed by running RAxML on the *x*-estimated MSA as input.

**Table 1.** Simulation study: model condition parameters and summary statistics

Model condition	Number of taxa	Model tree height	Insertion/ deletion	True alignment			MAFFT alignment			RAxML (MAFFT)
			Probability	ANHD	Gappiness	Length	Length	SP-FN	SP-FP	nRF
10.A	10	0.47	0.13	0.380	0.591	2466	1543	0.566	0.629	0.186
10.B	10	0.7	0.1	0.479	0.618	2691	1602	0.687	0.750	0.243
10.C	10	1.2	0.06	0.591	0.645	2832	1588	0.811	0.850	0.443
10.D	10	2	0.031	0.642	0.591	2490	1583	0.815	0.841	0.464
10.E	10	4.4	0.013	0.696	0.578	2390	1623	0.904	0.913	0.664
50.A	50	0.45	0.06	0.415	0.667	3070	2053	0.340	0.336	0.084
50.B	50	0.73	0.03	0.513	0.603	2525	1834	0.451	0.431	0.146
50.C	50	1.2	0.02	0.598	0.620	2646	1950	0.731	0.704	0.322
50.D	50	2	0.012	0.667	0.629	2720	2171	0.902	0.881	0.517
50.E	50	4.3	0.005	0.715	0.591	2474	2385	0.974	0.965	0.755
100.A	100	4	$1\times 10^{-5}$	0.454	0.331	1682	1533	0.054	0.046	0.075
100.B	100	7	$1\times 10^{-5}$	0.540	0.439	2263	1861	0.209	0.176	0.119
100.C	100	15	$5\times 10^{-5}$	0.646	0.571	2317	2418	0.680	0.603	0.470
100.D	100	25	$2\times 10^{-5}$	0.683	0.634	1837	2799	0.899	0.853	0.607
100.E	100	20	$4\times 10^{-5}$	0.672	0.614	2487	2701	0.848	0.796	0.661

*Note*: Model condition parameters consisted of the number of taxa, tree height and insertion/deletion probability. Each 10-taxon model condition is named 10.A through 10.E in generally increasing order of evolutionary divergence; the 50-taxon and 100-taxon model conditions are named similarly. The following average summary statistics are reported for true MSAs and MAFFT-estimated MSAs on each model condition (*n* = 20): ‘ANHD’ is the average normalized Hamming distance of a pair of aligned sequences in an MSA, ‘Gappiness’ is the proportion of an MSA matrix that consists of indels, ‘length’ is the number of MSA columns, and ‘SP-FN’ and ‘SP-FP’ are the proportions of nucleotide–nucleotide homologies that appear in the true alignment but not in the estimated alignment or vice versa, respectively. The average normalized Robinson–Foulds distance (‘nRF’) between the model tree and the RAxML(MAFFT)-inferred tree is also reported for each model condition (*n* = 20).

**Phylogenetic bootstrap method and other support estimation methods**. Bootstrap analyses were run using the bootstrap implementation in version 8.2.11 of the RAxML software. Each bootstrap support analysis utilized 100 bootstrap replicates; downstream re-estimation and support calculation steps were otherwise identical to RAWR. The Supplementary Material also includes additional supplementary experiments with other support estimation methods, including aLRT (Anisimova and Gascuel, 2006) and TBE (Lemoine *et al.*, 2018).

### 3.2 Performance study using simulated and empirical benchmarking data

**Simulated benchmarking datasets**. Our simulation study utilized model conditions and synthetic benchmarks from two previous studies (Liu *et al.*, 2012; Wang *et al.*, 2018). The model conditions exhibit varying dataset sizes and evolutionary divergence that are meant to capture a range of computational difficulty. We briefly recap the simulation procedures. First, model trees were sampled as follows. For the 10-taxon and 50-taxon simulations, INDELible version 1.03 (Fletcher and Yang, 2009) was used to sample non-ultrametric trees under a random birth process with branch lengths drawn uniformly at random from the open unit interval. For the 100-taxon simulations, random birth–death model trees were sampled using r8s version 1.7 (Sanderson, 2003). The 100-taxon model trees were then deviated from ultrametricity using Nakhleh *et al.* (2002)’s procedure with deviation factor *c *=* *2.0; the procedure was performed using a custom script (available at http://www.cs.utexas.edu/users/tandy/science-paper.html and https://github.com/tandyw/datasets/). Model trees were then rescaled to obtain total height specified by model condition parameter *h*. Then, nucleotide sequence evolution on model trees was simulated under a finite-sites models of nucleotide substitutions and sequence insertions/deletions with root sequence length of 1 kb. (As noted above, the sequence evolution model in our simulation study violates the i.i.d. site assumption.) The former consisted of the general time-reversible (GTR) model (Rodriguez *et al.*, 1990) with base frequency and substitution rate parameter settings based upon empirical NemAToL estimates from the study of Liu *et al.* (2012). INDELible (Fletcher and Yang, 2009) was used to simulate 10- and 50-taxon datasets under the GTR model and the indel model of Fletcher and Yang (2009). The 100-taxon simulations were performed using ROSE under the GTR model and an indel model with a ‘medium’ gap length distribution, as described by the earlier study of Liu *et al.* (2012). For each model condition, the simulation procedure was repeated to obtain 20 experimental replicates. Model condition parameters and summary statistics for simulated datasets are listed in Table 1.

All simulation study experiments in the main manuscript were independently performed in triplicate to help ensure reproducibility of study findings.

**Algorithm 1 RAWR phylogenetic support estimation**

1: **procedure** RAWRSupport(*A*, *T*, f(), g(), *γ*, *k_r_*) ▹ Input: MSA *A*, phylogenetic tree *T*, MSA method f(), ▹ phylogenetic tree estimation method g(), reversal probability *γ*, ▹ number of replicates *k_r _* ▹ Output: phylogenetic support estimates *ϵ*

2: reestimates = <>

3: **for** *i *=* *1 **to** *k_r_* **do**

4: *X_i_* = resampleRAWRReplicate(*A*)

5: reestimates.= $g(f(X_{i}))$

6: **for all** non-leaf edge *e* in *T* **do**

7: $\epsilon (e)=\text{proportion}\;\text{of}\;T_{i}\;\text{in}\;\text{list}\;\text{reestimates}\;$  that display bipartition corresponding to e

8: **return**(*ϵ*)

9: **procedure** resampleRAWRReplicate(*A*,*γ*)

10: *Y* = <>

11: select i∈[1,|A|] and walkDirection uniformly at random

12: **while **!converged(*Y*, *A*) **do**

13: *Y* .= *A*[*i*] ▹ add *i*th column of *A* to *Y*

14: **if** reversal(*γ*) ||  (i==1 && walkDirection is left)

15:     ||  (i==|A| && walkDirection is right)  **then**

16: reverse(walkDirection)

17: *i* = next column index after *i* in walkDirection order

18: **return**(unalign(*Y*)) ▹ unalign(*Y*) drops indels from *Y*

19: **procedure** converged(*Y*, *A*)

20: **return**(length(*Y*) ≥ length(*A*)) ▹ Sequence-length-based convergence criterion requires ▹ number of resampled sites ≥ input MSA length

**Empirical benchmarking datasets**. The empirical benchmarking data used in our study was obtained from the Comparative RNA Website (CRW) database (accessible at www.rna.icmb.utexas.edu) (Cannone *et al.*, 2002). The CRW rRNA datasets used in our study were provided with comprehensively curated multiple sequence alignments that were produced using intensive hybrid (i.e. automated and human) analysis of biomolecular sequence, structural and other information; the curated sequence alignments represent a ‘gold standard’ reference for benchmarking studies involving sequence alignment tasks (Liu *et al.*, 2009, 2012). The reference alignments were used to obtain reference trees, which consisted of MLE trees estimated on reference alignment. RAxML was used to perform these analyses using the same command as in the simulation study. As in earlier studies (Liu *et al.*, 2009, 2012; Mirarab *et al.*, 2015), our choice of reference tree is a practical one in the absence of ground truth.

Our simulation study model conditions best reflect non-coding nucleotide sequence evolution, and our empirical study focuses on intronic rRNA datasets for experimental consistency. Similar to the simulation study, we selected datasets with a range of evolutionary divergence and dataset sizes up to 250 sequences. Sequences with greater than 99% missing data were omitted from analysis. Table 2 and Supplementary Figure S3 provide summary statistics and other information on the empirical datasets.

**Table 2.** Empirical study: summary statistics.

Dataset	Number of taxa	Reference alignment			MAFFT alignment			RAxML (MAFFT)
		ANHD	Gappiness	Length	Length	SP-FN	SP-FP	nRF
IGIA	110	0.606	0.915	10368	6065	0.732	0.780	0.645
IGIB	202	0.579	0.910	10633	7070	0.825	0.863	0.678
IGIC2	32	0.533	0.700	4243	3530	0.691	0.716	0.517
IGID	21	0.719	0.782	5061	3063	0.874	0.905	0.778
IGIE	249	0.451	0.838	2751	2847	0.406	0.389	0.585
IGIIA	174	0.668	0.814	6406	6945	0.817	0.800	0.450

*Note*: The empirical datasets used in our performance study were obtained from the Comparative RNA Website (CRW) database (Cannone *et al.*, 2002). (See Materials and Methods section for more details.) The curated alignment and a maximum likelihood tree estimated on the curated alignment were used as the reference alignment and tree for benchmarking purposes. We report the following summary statistics for each reference alignment (*n* = 1): ‘ANHD’ or average normalized Hamming distance, ‘Gappiness’, and ‘length’; we also report the MAFFT-estimated MSA ‘length’ and ‘SP-FN’ and ‘SP-FP’ errors with respect to the reference alignment, as well as the ‘nRF’ or normalized Robinson–Foulds distance between the RAxML(MAFFT) tree and the reference tree. Summary statistic calculations and descriptions are otherwise identical to Table 1.

All empirical benchmarking experiments in the main manuscript were independently performed in triplicate to help ensure reproducibility of study findings.

**Performance criteria used in benchmarking evaluations**. Phylogenetic support estimation methods were evaluated based on type I and type II error of estimated phylogenetic support for a phylogenetic tree estimate with respect to a reference tree (i.e. the model tree for each simulated dataset and the reference tree for each empirical dataset). We used precision-recall (PR) curves and area under PR curves (PR-AUC) to evaluate both types of error and tradeoffs between them. We note that the phylogenetic support estimation problem (and, more generally, classical estimation of confidence intervals in statistics) requires an original estimate for annotation; a two-phase method was used for this purpose (see ‘Methods for MSA and phylogenetic estimation/re-estimation’ text above). A phylogenetic support estimation method was then run to annotate each branch of the estimated tree with a phylogenetic support value and thereby place confidence intervals on the estimated tree topology. For this reason, the confusion matrix for the PR curves was formed from the following four classes: true positives (TP) consist of bipartitions of the estimated tree that have support value greater than or equal to a given threshold and appear in the reference tree, false positives (FP) consist of bipartitions of the estimated tree that have support value greater than or equal to a given threshold but do not appear in the reference tree, false negatives (FN) consist of bipartitions of the estimated tree that have support less than a given threshold but appear in the reference tree, and true negatives (TN) consist of bipartitions of the estimated tree that have support less than a given threshold and do not appear in the reference tree. The PR curve plots true positive rate ($\frac{\left|\text{TP}\right|}{\left|\text{TP}|+|\text{FN}\right|}$) versus precision ($\frac{\left|\text{TP}\right|}{\left|\text{TP}|+|\text{FP}\right|}$), and varying the threshold for confusion matrix calculations yields different points along the curve. We used custom scripts and the scikit-learn Python library (Pedregosa *et al.*, 2011) to calculate the curves and AUC quantities.

We also compared phylogenetic support estimation methods based on serial computational runtime and peak main memory usage. All experiments were conducted on computing facilities in the Michigan State University High Performance Computing Center. We used compute nodes in the intel16-k80 cluster, each of which had a 2.4 GHz 14-core Intel Xeon E5-2680v4 processor.

### 3.3 Re-analysis of Darwin’s finches dataset

**Genomic sequences and sequence alignments**. As part of our empirical study, we re-analyzed genomic sequence data from Lamichhaney *et al.* (2015)’s study of Darwin’s finches. The raw NGS dataset was obtained and processed into a multi-locus sequence dataset using the steps listed in the Supplementary Methods text (see Supplementary Material). A concatenated genomic sequence alignment *A* was produced by concatenating multiple sequence alignments across all loci in the multi-locus sequence dataset. The $i^{\text{th}}$ alignment *a_i_* served as partition *p_i_* in the concatenated alignment and the following analyses.

**Species tree estimation**. To obtain an annotation phylogeny for the phylogenetic support estimation methods under study, a species tree was estimated using maximum likelihood estimation (MLE) on the concatenated and partitioned genomic sequence alignment *A*. In terms of sequence length, the resulting aligned dataset *A* was larger than all of the other datasets in our study by multiple orders of magnitude. We therefore utilized ExaML (Kozlov *et al.*, 2015) to perform parallelized computation on a high-performance computing cluster as a means to cope with the increased computational requirements of phylogenetic MLE. We obtained an initial tree for ExaML’s local search heuristics by performing maximum parsimony optimization using RAxML version 8.2.9. ExaML version 3.0.21 was used to perform phylogenetic MLE on the concatenated and partitioned alignment *A*.

**Phylogenetic support estimation using bootstrap resampling**. The standard bootstrap method was used to sample 100 bootstrap replicates from the concatenated and partitioned genomic sequence alignment *A*. We used RAxML version 8.2.9 command to conduct bootstrap resampling. To avoid degeneracy with learning substitution model parameters, each bootstrap replicate was filtered to only retain partitions where all four nucleotides were present. Each bootstrap replicate’s concatenated and partitioned MSA was then converted into binary ExaML format using the command listed in the Supplementary Methods text.

An MLE tree was then re-estimated on each bootstrap replicate’s concatenated and partitioned MSA. RAxML and ExaML were used to perform phylogenetic MLE with the same software versions and commands as in the original species tree estimation step. The re-estimated trees were used to estimate phylogenetic bootstrap support for the species tree that we estimated using the above MLE analysis.

**Phylogenetic support estimation using RAWR**. RAWR was run using default settings to resample each estimated alignment *a_i_* and thereby obtain 100 resampled replicates $\left\{b_{ij}\right\}$ for 1≤j≤100. To obtain a single concatenated and partitioned replicate dataset for re-estimation purposes, we constructed the supermatrix *A_j_* to be the concatenation of resampled partition replicates $\left\{b_{ij}\right\}$ where all four nucleotides were present, as in the bootstrap analyses. Phylogenetic re-estimation was then conducted on each RAWR replicate dataset *A_j_* in a manner identical to the bootstrap analyses; the same software, software versions and commands were used. As in the bootstrap analyses, the re-estimated RAWR trees were used to calculate support for the MLE species tree using the same software and software commands. We used Dendroscope (Huson and Scornavacca, 2012) to visualize phylogenetic support estimates on the species tree.

## 4 Results

### 4.1 Simulation study

**Performance comparison of RAWR versus bootstrap**. We compared the performance of RAWR versus bootstrap on the simulation study datasets, where MAFFT and/or RAxML(MAFFT) were used to estimate/re-estimate MSAs and phylogenetic trees. The type I and type II error of the different methods were evaluated based on PR-AUC, as shown in Table 3.

**Table 3.** Simulation study: PR-AUC comparison of bootstrap, RAWR-reduced, and RAWR methods for phylogenetic support estimation.

Model condition	PR-AUC			Corrected q-value
	Bootstrap	RAWR-reduced	RAWR	
10.A	0.951	**0.989**	**0.996**	$8.2\times 10^{-3}$
10.B	0.920	0.978	**0.990**	$4.2\times 10^{-3}$
10.C	0.784	0.927	**0.977**	$4.2\times 10^{-3}$
10.D	0.822	0.950	**0.968**	$4.2\times 10^{-3}$
10.E	0.679	**0.976**	0.925	$1.5\times 10^{-4}$
50.A	**0.988**	**0.993**	**0.997**	$4.3\times 10^{-3}$
50.B	0.970	**0.990**	**0.994**	$5.4\times 10^{-4}$
50.C	0.900	**0.980**	**0.989**	$4.9\times 10^{-6}$
50.D	0.798	**0.981**	**0.988**	$<10^{-10}$
50.E	0.663	**0.990**	**0.997**	$<10^{-10}$
100.A	**0.997**	**0.990**	**0.993**	n.s.
100.B	**0.990**	**0.986**	**0.991**	n.s.
100.C	0.828	0.971	**0.982**	$7.2\times 10^{-9}$
100.D	0.735	0.973	**0.983**	$<10^{-10}$
100.E	0.695	0.975	**0.986**	$<10^{-10}$

*Note:* MAFFT and RAxML(MAFFT) were used to perform MSA and tree estimation/re-estimation, respectively. We report each method’s aggregate PR-AUC across all replicate datasets for a model condition (*n* = 20). For each model condition, the top PR-AUC values within an absolute difference of 0.01 are shown in bold. Statistical significance of PR-AUC differences between RAWR and bootstrap were evaluated using a one-tailed pairwise *t*-test and a multiple test correction was performed using the method of Benjamini and Hochberg (1995). Corrected q-values are reported (*n* = 20).

RAWR consistently returned comparable or better PR-AUC compared to bootstrap. RAWR improvements over bootstrap were statistically significant using pairwise *t*-tests with Benjamini Hochberg correction (Benjamini and Hochberg, 1995) (*n *=* *20 and α=0.05) on all model conditions with just two exceptions—the two least divergent 100-taxon model conditions, where all methods returned comparable PR-AUC (within 0.01 of the best method).

RAWR’s PR-AUC advantage over bootstrap tended to grow as model conditions grew larger and/or more divergent. This suggests that RAWR support estimates offer better type I and type II error on more challenging datasets. The maximum absolute PR-AUC improvement of RAWR over bootstrap was 0.334 , and the average across all model conditions was 0.136. A difference between the methods is that RAWR incorporates re-estimation of both MSAs and phylogenetic trees, but bootstrap only incorporates re-estimation of trees. This is because it doesn’t make sense to re-estimate an MSA on a bootstrap replicate, since the lack of neighbor preservation could lead to a possible loss of sequence homology.

We also compared serial runtime and peak memory usage for all methods (Supplementary Figure S1). Compared to the bootstrap method, the non-bootstrap methods require an additional alignment re-estimation step on each resampled replicate. This key difference resulted in multiple factors of greater runtime for the former as compared to the latter, where all methods utilized the same amount of resampling replication (i.e. 100 replicates). Overall, absolute runtimes were relatively short on 10-taxon model conditions—on the order of minutes for bootstrap and RAWR—but grew quickly as the number of taxa increased. On the most divergent 100-taxon model condition, analyses took between half a day and multiple days to finish. We attribute observed runtime trends to the computational complexity of MSA estimation and phylogenetic MLE problems (Roch, 2006; Wang and Jiang, 1994).

A comparison of peak memory usage resulted in qualitatively similar method rankings, although the absolute differences were not as large as in the case of the runtime comparisons. Peak memory usage was modest in our study—amounting to just a few hundred MiB, an amount well within the scope of modern PC specifications. The bootstrap method typically utilized the least main memory compared to the other methods under study. As in other related studies (Liu *et al.*, 2012; Mirarab *et al.*, 2015), we anticipate that, due to the computational difficulty of the applications in our study, memory limitations will quickly become a major bottleneck as dataset sizes increase.

Overall, the simulation study experiments suggest that RAWR offers a performance advantage in terms of type I and type II error versus the state-of-the-art; this improvement come at the cost of increased time and memory usage relative to a standard bootstrap analysis.

**RAWR support estimation using reduced resampling replication**. Our performance study also included a conservative comparison where we reduced the number of resampled replicates in our RAWR methods’ analyses by an order of magnitude (i.e. 10 resampled replicates as opposed to the 100 resampled replicates used by the other phylogenetic support estimation methods). We refer to the resulting method as ‘RAWR-reduced’. Despite the reduction of resampled data, RAWR-reduced returned comparable or better PR-AUC compared to bootstrap, with greater PR-AUC improvements occurring on larger and/or more divergent model conditions. The PR-AUC returned by RAWR-reduced was comparable to RAWR, amounting to an average absolute difference of 0.007 across all model conditions (Table 3). On average for each model condition, the RAWR-reduced analyses had slightly larger serial runtime compared to the bootstrap method, but were smaller than RAWR by multiple factors; both RAWR and RAWR-reduced had similar peak memory usage (Supplementary Figure S1). Thus, where computational efficiency is a concern, the use of reduced resampling replication in RAWR analyses allows a tradeoff between type I/II error and computational efficiency, without too great of a penalty for the former.

### 4.2 Empirical study

*Performance benchmarking using CRW datasets with reference MSAs*. We also evaluated RAWR versus bootstrap based on their PR-AUC on the empirical datasets (Table 4). The PR-AUC comparison outcomes were similar to those seen in the simulation study. RAWR outperformed the bootstrap method on all empirical benchmarks, with an average absolute improvement of 0.105.

**Table 4.** Empirical study: PR-AUC comparison of bootstrap and RAWR methods for phylogenetic support estimation

Dataset	PR-AUC	
	Bootstrap	RAWR
IGIA	0.725	**0.804**
IGIB	0.629	**0.695**
IGIC2	0.778	**0.957**
IGID	0.670	**0.884**
IGIE	0.772	**0.808**
IGIIA	0.830	**0.884**

*Note*: MAFFT and RAxML(MAFFT) were used to perform MSA and tree estimation/re-estimation, respectively. For each empirical dataset, the top PR-AUC value is shown in bold.

Three differences between the empirical study and simulation study are worth noting. Reference trees in the empirical study are not the same as ground truth in the simulation study. The former consisted of MLE-inferred trees on highly accurate curated alignments, which in turn are expected to be highly accurate (but not perfectly accurate). Also, by necessity, the number of benchmarking datasets differed between the simulation and empirical study. This is due to the large amount of effort required to curate reference alignments for empirical datasets. Finally, compared to other CRW datasets, intronic rRNA markers are closest to the simulation study model conditions, but still not exactly the same. The former involve secondary structure evolution, strong selective pressures, and other evolutionary and biophysical constraints that are not accounted for by the estimation/re-estimation methods in our study. This plays a role in the somewhat lower PR-AUC values observed in the empirical study, as compared to the simulation study.

*Re-analysis of Darwin’s finches study dataset*. As shown in Figure 3, the concatenated MLE analysis in our study returned a phylogenetic tree estimate that was topologically identical to study of Lamichhaney *et al.* (2015) [see Figure 1 panel b in Lamichhaney *et al.* (2015)], with one exception. The two topologies disagreed only on a single internal branch in the tree finches clade.

The same tree finches clade yielded a major discrepancy between the two support estimation methods under study. RAWR returns lower support than bootstrap in the tree finches clade. The latter returns 100% on all non-trivial internal branches while the former does not: RAWR estimated 46% support on the parent edge for the most recent common ancestor (MRCA) of *Camarhynchus psittacula*, *Camarhynchus heliobates* and *Camarhynchus pallidus*, and 68% support on the parent edge for the MRCA of the previous three *C.* species as well as *Camarhynchus pauper*. This is noteworthy since the clade involved is the only one that has different bipartitions compared to the published tree in the original study of Lamichhaney *et al.* (2015). Our findings suggest that the phylogenetic relationships inferred in this clade are more uncertain than elsewhere in the species phylogeny once MSA quality is factored into phylogenetic support estimates.

Smaller discrepancies were also noted in the non-sharp-beaked ground finches clade—at most a 7% difference. Otherwise, both methods closely agreed on estimated bipartition support in the rest of the species phylogeny, with an average (and median) support difference of 0.6% (and 0%).

We note that our empirical dataset re-analysis had one major difference compared to the rest of our study: the former analyzed a genome-scale multi-locus dataset and single locus datasets were used elsewhere in our study. Consistent with Lamichhaney *et al.*’s study methodology and standard practice in many current phylogenomic studies, partitioned MLE analyses were used on the concatenated multi-locus dataset.

## 5 Discussion

Our findings reflect some basic observations about random walk resampling. The neighbor preservation property is a critical difference between RAWR resampling and bootstrap resampling: the former has it, while the latter does not. The neighbor preservation property helps to ensure that meaningful sequence homology is retained within a resampled replicate and subsequent alignment/tree re-estimation is well-defined.

We note that, compared to SERES, RAWR is closer in design to the bootstrap. There is no need for an anchor selection step and its additional parameters. A simpler formulation should be more tractable to both experimental and theoretical study.

Random walk reversals allow for more replicates beyond the two possible via mirrored inputs, and increased resampling generally improves support estimation. However, the increased resampling replication comes at a price. Reversal introduces a form of ‘noise injection’. Ideally, if perfect sequence alignments were attainable, all nucleotides near a breakpoint would be correctly aligned and re-estimated homologies would be ‘synchronized’ in terms of sequence homology; in practice, incorrect subsequence alignments near a reversal breakpoint introduce the possibility for ‘de-synchronized’ re-estimations (i.e. aligning nucleotide pairs for which homology is not well-defined). Too low of a reversal probability effectively limits the number of distinct resampled replicates that are possible, but too high of a reversal probability results in more ‘noise’ that can impact downstream re-estimation. Type I/II error of downstream support estimates are somewhat impacted by this choice, although experiments suggest that a reasonable choice is to err on the smaller side for *γ* settings.

There is another connection between RAWR resampling and bootstrap resampling. Higher *γ* values also have the effect of reducing sequential dependence in RAWR replicates. Lack of sequential dependence is a primary characteristic of bootstrap resampling. These assertions can be seen via some thought experiments. The following two cases bracket two endpoints along a continuum of sequential dependence: (i) RAWR with *γ *= 0 is equivalent to mirrored inputs but with a random start point and reflection at start/ends of sequences and (ii) RAWR with γ=0.5 is a first-order Markovian process, as discussed in Wang *et al.* (2018). Intermediate cases fall along a continuum specified by RAWR with 0<γ<0.5 and require a second-order Markovian process, where a smaller choice of *γ* effectively preserves longer-range sequential dependence in resampled RAWR replicates. We view γ≈0.5 to be very large and, as mentioned above, smaller values are likely to be more practical for most applications. Results from our experiments with increasing RAWR reversal probability *γ* are consistent with this thinking.

The resampled site distribution for an *individual RAWR replicate* depends upon the choice of reversal probability *γ*. This can be seen by considering a simpler ‘unidirectional’ version of a RAWR walk on an infinitely long input sequence of observations, where resampling terminates immediately with probability *γ* in lieu of a reversal event; site resampling will be geometrically distributed with expected replicate length $\frac{1}{\gamma }$. Alternatively, we can consider an extreme case: RAWR with *γ *= 1 is equivalent to sampling a single site many times and should result in catastrophic data loss. Taken together, these observations indicate that an individual RAWR replicate’s resampling can be biased around the initial start position. Furthermore, reflection is certain at the start and end of the input sequence of observations, which can also introduce RAWR resampling bias.

However, the above observations do not apply to a sufficiently large *set of RAWR replicates* in aggregate, since RAWR start sites are chosen uniformly at random. Observed sampling frequency histograms for RAWR resampling (Supplementary Figure S2) are consistent with this thinking. RAWR support estimation therefore mitigates the impact of bias since it utilizes many resampled RAWR replicates and re-estimations to produce a single set of support estimates.

Extensions to the standard RAWR resampling procedure can help to address theoretical resampling bias of individual replicates. We include supplementary experiments with one such extension that replaces random reversals with random ‘teleportation’: with probability *γ*, an in-progress random walk chooses the next start site and walk direction uniformly at random (Supplementary Algorithm 2). The alternative resampling procedure can be seen as an intermediate between standard bootstrap resampling and standard RAWR resampling that combines aspects of both. The resulting phylogenetic support estimates yielded similar PR-AUC performance in supplementary experiments (Supplementary Figure S9).

We note that theoretical guarantees about phylogenetic bootstrap estimation typically ignore the issue of alignment error and effectively assume that the annotation MSA is perfectly accurate. But annotation MSAs are never totally correct in practice. Therefore, RAWR’s performance improvement must be due in part to annotation MSA inaccuracy. We hypothesize that data augmentation using RAWR replicates allows re-estimation to effectively explore more of the underlying search space for the MSA and tree estimation problems. Confirmation of this hypothesis would reinforce previous studies that demonstrated the major impact of MSA quality on phylogenetic reconstruction accuracy (Liu *et al.*, 2012, 2009; Nelesen *et al.*, 2008).

We view the new random walk resampling and re-estimation techniques to be a next step toward the ultimate goal of fully addressing i.i.d. assumptions in biomolecular sequence analysis and other topics, with the caveat that the contribution is necessary but not sufficient. As applied in this study, RAWR resampling and re-estimation focuses on sequence dependence due to insertion and deletion processes. But sequence dependence arising from other factors that are not accounted for in this study, and, at least in its current form, RAWR effectively retains the same simplifying assumptions about these other factors as other state-of-the-art methods. There is still more progress to be made. Below, we highlight future work that can further close the gap, including possible extensions of the RAWR framework.

The concatenated MLE analysis in our study reproduced the same species tree topology as in study of Lamichhaney *et al.* (2015), with the exception of one internal branch in the tree finches clade. These species tree topologies have multiple important differences compared to classical taxonomy based on morphological and mitochondrial data analysis. Lamichhaney *et al.* provide evidence that genetic drift/incomplete lineage sorting (ILS) and interspecific gene flow are two important factors behind these topological differences. We found major differences in support estimation on the discordant branch and one other branch in tree finch clade, and minor differences in the non-sharp-beaked ground finch clade.

Our findings shed new light into the biological significance of phylogenetic uncertainty in the evolutionary history of Darwin’s finches. Sequence insertion/deletion processes and MSA quality are also important factors in species phylogeny reconstruction for the taxa under study. The combination of sequence insertion and deletion processes with other complex evolutionary processes (i.e., genetic drift, ILS and gene flow) likely played an important role during the rapid adaptive radiation of Darwin’s finches.

Our study focused on traditional phylogenetic estimation methods that do not account for local phylogenetic discordance in multi-locus sequence data. We hypothesize that random walk resampling and re-estimation will yield similar benefits in the context of phylogenomic inference/learning using multi-locus/genomic sequence data. Utilizing coalescent-based statistical methods (Hejase *et al.*, 2018; Mirarab *et al.*, 2014) will enable direct evaluation of the role of different genome evolution processes (genetic drift/ILS, gene flow, sequence insertion/deletion, etc.) in shaping the species phylogeny under study.

## 6 Conclusion

In this study, we introduced RAWR, a new non-parametric resampling technique. We applied RAWR to the widely studied task of phylogenetic support estimation. On simulated and empirical benchmarks spanning a range of dataset sizes and sequence divergence, we found that RAWR support estimates had comparable or often better type I and type II error compared to other state-of-the-art methods. RAWR resampling/re-estimation had increased computational overhead compared to a standard bootstrap-based pipeline. The tradeoff between accuracy and computational runtime can be offset through reduced resampling replication. RAWR also revealed the impact of sequence insertion and deletion processes on phylogenetic estimation uncertainty for Darwin’s finches, a classical model of rapid adaptive radiation.

We conclude with thoughts on directions for future research. First, feature-aware and application-aware resampling and re-estimation will help narrow the gap toward fully accounting for sequence dependence in biomolecular sequence analysis. Examples include random walk resampling procedures that account for codon structure in coding DNA sequences, secondary structure in RNA sequences and tertiary structure in amino acid sequences; re-estimation that accounts for biomolecular structure and evolutionary conservation in RNA sequences would help ameliorate model mis-specification in the empirical study. Second, non-parametric resampling techniques like RAWR typically require fewer assumptions and are not defined on or constrained to a specific application, unlike parametric and semi-parametric resampling methods. RAWR can therefore be easily applied to any problem with sequence inputs or sequential dependence. One important example is statistical inference of species trees under non-i.i.d. models of sequence evolution (Thorne *et al.*, 1992; Wang and Liu, 2016) that move beyond the traditional focus on i.i.d. point mutation models and properly leverage the tantalizing phylogenetic signal left by sequence insertion and deletion processes (Warnow, 2012). We envision many future applications in computational biology and bioinformatics and beyond, including RNA secondary structure and other biomolecular structure prediction, machine learning using temporal models (Breiman, 1996), and many more.

## Supplementary Material

btab263_Supplementary_Data
