# Supplementary material for: Build a better bootstrap and the RAWR shall beat a random path to your door: phylogenetic support estimation revisited
Source: Bioinformatics. 2021 Jul 12;37(Suppl 1):i111–9. doi: 10.1093/bioinformatics/btab263 (PMC8336443; doi:10.1093/bioinformatics/btab263)
Supplement: btab263_Supplementary_Data [file btab263_supplementary_data.pdf]

Supplementary Material – Build a Better Bootstrap and the RAWR Shall Beat a Random Path to Your Door: Phylogenetic Support Estimation Revisited

Wei Wang [1], Ahmad Hejasebazzi [1], Julia Zheng [1,2], and Kevin J. Liu [1,2,3*]
[1]Department of Computer Science and Engineering,
[2], Ecology, Evolution, and Behavior Program,
[3]Genetics and Genome Sciences Program,
Michigan State University, East Lansing, MI 48824, USA.
*To whom correspondence should be addressed.

1 Supplementary Methods

Darwin's finches dataset processing. Our study re-analyzed genomic sequence data that was originally studied by Lamichhaney *et al.* [5]. We began by downloading the original Illumina HiSeq2000 paired-end read data from the NCBI SRA database (accession number PRJNA263122 at http://www.ncbi.nlm.nih.gov/sra). One sample was randomly selected for each species, resulting in a dataset with 25 samples (accession numbers SRR1607296, SRR1607504, SRR1607439, SRR1607359, SRR1607385, SRR1607440, SRR1607547, SRR1607403, SRR1607458, SRR1607472, SRR1607551, SRR1607494, SRR1607399, SRR1607462, SRR1607343, SRR1607534, SRR1607406, SRR1607485, SRR1607508, SRR1607543, SRR1607365, SRR1607420, SRR1607466, SRR1607529, and SRR1607480). We also downloaded the assembled whole-genome sequence and gene annotations for the medium ground finch *Geospiza fortis* (available from the GigaDB database at http://gigadb.org/dataset/100040).

NGS read alignment, quality filtering, and variant calling steps were based on the study of [5]. First, paired-end reads for each sample were aligned to the *G. fortis* reference genome using BWA version 0.7.17 with default parameters [8]. Quality filtering, post-processing, and variant calling tasks were then performed using SAMtools [9]. Identified variants consisted of SNPs and short indel polymorphisms.

To obtain haplotype sequences, bi-allelic SNPs were phased using fastPHASE version 1.4.8 [15]. The phased calls for bi-allelic SNPs were then combined with genotypic data for the

non-bi-allelic SNPs and short indel polymorphisms, where heterozygous allele calls for the latter were treated as missing data.

A multi-locus sequence dataset was constructed by delineating loci based on gene annotations provided with the reference genome, where each locus corresponded to either an annotated gene, an intergenic region between annotated genes on a scaffold, or a scaffold with no annotated genes. A multiple sequence alignment was then estimated for each locus using MAFFT version 7.222 [3] with default options. Finally, loci were filtered based on an estimated MSA length criterion of at least 30 bp and at most 1.5 Mb. The final multi-locus sequence dataset consisted of 13,321 loci corresponding to annotated genes, 15,275 loci corresponding to intergenic regions between annotated genes on a scaffold, and 6,376 loci corresponding to scaffolds that lacked annotated genes.

Experiments using ClustalW to estimate/re-estimate MSAs. To explore the impact of alignment quality on downstream phylogenetic inference and support estimation, we also included ClustalW in our performance study since it was one of the first popular MSA methods and has since become a mainstay throughout computational biology and bioinformatics. Our ClustalW analyses were run using the version 2.1 software with default settings. Summary statistics for the ClustalW-estimated MSAs are shown in Supplementary Table S2.

Experiments on 10-taxon model conditions with a long gap length distribution. Our simulation study also included a performance evaluation using additional model conditions from [18] that utilized the "long" gap length distribution from [11], rather than the "medium" gap length distribution used elsewhere in our simulation study. The simulation and experimental procedures were exactly the same across all model conditions in our simulation study. For reference, Table S1 lists model condition parameters and summary statistics for the long-gap-length simulations (reproduced from [18]).

GUIDANCE2 experiments. Our supplementary experiments also include a performance comparison using GUIDANCE2 [16], a method for placing confidence intervals on estimated multiple sequence alignments. GUIDANCE2 augments Landan and Graur [6]'s "mirrored inputs" idea with progressive MSA-specific parametric resampling techniques. GUIDANCE2 was run with default settings. For each dataset, GUIDANCE2 was used to resample 100 replicates and re-estimation was performed on each resampled replicate using an identical procedure as in the RAWR and bootstrap analyses. The support calculation made use of the RAxML software and the same command as used elsewhere in our study. Although Landan and Graur [6] originally focused on MSA estimation and GUIDANCE2 was originally developed for MSA confidence interval placement, it is natural to consider the impact of MSA quality on downstream inference tasks. As we demonstrate in our performance study, a new application of these parametric and semi-parametric techniques beyond their originally intended use can bring value.

aLRT experiments. We used the aLRT [1] implementation in PhyML version 3.0 [2] to run two aLRT analyses. (1) We ran a parametric aLRT support analysis under the general time reversible (GTR) nucleotide substitution model [13], where a "free" phylogenetic tree

Supplementary Table S1: **Long-gap-length model conditions: parameter values and summary statistics.** Our simulation study included additional 10-taxon model conditions that utilized the long gap length distribution from the study of Liu *et al.* [11]. The model parameters consisted of model tree height and insertion/deletion probability, and each model condition corresponds to a distinct set of model parameter values. The long-gap-length model conditions are named 10.long.A through 10.long.E in order of generally increasing sequence divergence. The following table columns list average summary statistics for each model condition ($n = 20$). "NHD" is the average normalized Hamming distance of a pair of aligned sequences in the true alignment. "Gappiness" is the percentage of true alignment cells which consists of indels. "True align length" is the length of the true alignment. "Est align length" is the length of the MAFFT-estimated alignment [3] which was provided as input to the support estimation methods. "SP-FN" and "SP-FP" are the proportion of homologies that appear in the true alignment but not in the MAFFT-estimated alignment and vice versa, respectively. The table and caption are reproduced from [18].

**Model**	**Tree**	**Insertion/deletion**	**True align**			**Est alignment**		
**condition**	**height**	**probability**	**NHD**	**Gappiness**	**length**	**length**	**SP-FN**	**SP-FP**
10.long.A	0.4	0.13	0.276	0.440	1804.8	1433.7	0.272	0.315
10.long.B	0.7	0.1	0.363	0.481	1926.7	1447.8	0.381	0.426
10.long.C	1	0.06	0.455	0.456	1853.5	1413.3	0.510	0.537
10.long.D	1.6	0.031	0.542	0.432	1754.1	1403.1	0.725	0.729
10.long.E	4.3	0.013	0.660	0.445	1811.0	1560.1	0.899	0.897

topology was estimated alongside GTR substitution model parameters and branch lengths. The following command was used to run the analysis:

```
phyml -i <input alignment file> -d nt -m GTR -b -1 -o tlr
```

(2) We also ran a fixed-topology analysis, where the fixed topology consisted of the annotation topology that was estimated by RAxML. The fixed-topology analysis included estimation of branch lengths and GTR substitution rates/frequencies using PhyML. The following command was used to run the second analysis:

```
phyml -i <input alignment file> -d nt -m GTR -b -1 -o lr
      -u <user-specified Newick tree file
          with RAxML-estimated annotation tree>
```

TBE experiments. TBE [7] was run using the same annotation MSA, annotation tree, and re-estimated trees (i.e., re-estimated trees generated by either bootstrap resampling and re-estimation or RAWR resampling and re-estimation) as in the rest of our performance study. The equivalent inputs enable a comparison across TBE and other methods in our study. The TBE analyses were run using Booster v. 0.1.2 with the following command:

```
booster_linux64 -a tbe -i <annotation tree file in Newick format>
                -b <re-estimated tree file>
                -@ <number of parallelized threads> -o <output file>
```

Experiments with alternative random walk resampling procedure. We also eval-

uated an alternative random-walk-based phylogenetic support estimation procedure. The alternative procedure replaced random reversals in the RAWR resampling procedure with random "teleportation", resulting in the resampling algorithm shown in Algorithm 2. We refer to the alternative method as "RAWR+teleport" for this reason.

Algorithm 2 RAWR+teleport resampling procedure

procedure RESAMPLEWITHRAWR+TELEPORT(A,γ)
    $Y = <>$
    select $i \in [1, |A|]$ and walkDirection uniformly at random
    while !converged(Y,A) **do**
        Y .= $A[i]$ ▷ add ith column of A to Y
        if teleport(γ) **then** ▷ Biased coin flip with bias γ
            select $i \in [1, |A|]$ and walkDirection uniformly at random
        else
            if (i == 1 && walkDirection is left) || (i == $|A|$ && walkDirection is right) **then**
                reverse(walkDirection)
            i = next column index after i in walkDirection order
    return(unalign(Y)) ▷ unalign(Y) drops indels from Y

Software commands used in performance study involving simulated and empirical benchmarking data. The following command was used to perform MSA estimation/re-estimation using MAFFT [3] version 7.222:

```
mafft <sequence file>
  > <estimated alignment file>
```

ClustalW version 2.1 was run using the following command to estimate/re-estimate MSAs:

```
clustalw2 -INFILE=<resampled sequence file>
  -ALIGN -TYPE=dna
  -outfile=<estimated alignment file>
  -output=FASTA
```

RAxML [17] analyses (other than bootstrap analyses) were performed with RAxML version 8.2.11 and the following command:

```
raxmlHPC -s <estimated alignment file>
  -n <name> -m GTRGAMMA -p <random number>
  -# 10
```

Bootstrap analyses were run using RAxML version 8.2.11 and the following command: The analyses were run using the following command:

```
raxmlHPC -s <estimated alignment file>
  -n <name> -m GTRGAMMA
  -p <random number> -b <random number>
```

```
  -# <number of sample trees>
```

The 100-taxon model trees in our simulation study were sampled using r8s [14] version 1.7 and the following script:

```
begin r8s;
simulate diversemodel=bdback seed=<random seed>
  nreps=20 ntaxa=<10 or 50> T=0;
describe tree=0 plot=chrono_description;
end;
```

Software commands used in experiments on Darwin's finches dataset. Multiple sequence alignments were converted from FASTA format into a compact binary format required by ExaML [4], a phylogenetic MLE software package tailored to high-performance scientific computing environments:

```
parse-examl -s <alignment file> -m DNA
  -q <partition file> -n <sample num>
```

To obtain a starting tree for ExaML MLE, we used RAxML version 8.2.9 and the following command to perform maximum parsimony optimization:

```
raxmlHPC-AVX -y -s <alignment file> -m GTRGAMMA
  -n <output file suffix/replicate ID>
  -p <random seed> -q <partition file>
```

ExaML MLE analyses were performed using version 3.0.21 of the ExaML software and the following command:

```
mpirun -np <number of threads> examl-AVX
  -t <start tree file> -m GAMMA
  -s <binary file> -n <output file suffix>
```

Bootstrap resampling was conducted using RAxML version 8.2.9 and the following command:

```
raxmlHPC-SSE3 -# 100 -b <random seed> -f j
  -m GTRGAMMA -s <concatenated alignment file>
  -q <partition file> -n <output file suffix>
```

Given the annotation tree and a set of bootstrap trees that were re-estimated on the resampled bootstrap replicates, the following RAxML command was used to calculate phylogenetic bootstrap support:

```
raxmlHPC -f b -m GTRGAMMA -t <tree file>
  -z <sampled trees> -n <name>
```

Supplementary Table S2: **Simulation study: summary statistics for ClustalW alignments and RAxML(ClustalW) trees on 10-taxon model conditions.** Table layout and description are otherwise identical to Table 1.

Model condition	ClustalW alignment length	SP-FN	SP-FP	RAxML (ClustalW) nRF
10.A	1216	0.670	0.747	0.207
10.B	1236	0.726	0.809	0.236
10.C	1184	0.835	0.889	0.379
10.D	1171	0.828	0.874	0.500
10.E	1163	0.901	0.926	0.650

2 Supplementary Results and Discussion

RAWR support estimation using alternative MSA/tree estimation/re-estimation methods. As in other performance studies of MSA and phylogenetic tree estimation from unaligned sequence inputs [10, 11], we found that MAFFT generally produced more accurate alignments than ClustalW on the 10-taxon model conditions, although this accuracy improvement did not translate directly to more accurate downstream phylogenetic inference (Table 1 and Supplementary Table S2).

Despite this, RAWR returned comparable PR-AUC regardless of which of the two MSA methods were used on the 10.A and 10.B model conditions. On the more divergent 10.C through 10.E model conditions, RAWR returned respective PR-AUC improvements of 0.011, 0.024, and 0.039 when using ClustalW and RAxML(ClustalW) for estimation/re-estimation, rather than MAFFT and RAxML(MAFFT) (Table S3). Our finding suggests that RAWR support estimation is robust to annotation MSA/tree quality, and hints at an even stronger result: neighbor-preserving random walks may yield better support estimates where computational problems are more difficult and estimation uncertainty is therefore greater.

RAWR support estimation using alternative choices for reversal probability γ. On each 10-taxon model condition except for the 10.C model condition, RAWR returned similar PR-AUC as the reversal probability γ was increased from 0.001 up until a critical threshold; PR-AUC then dropped as γ increased past the threshold (Table S4). The exact threshold varied somewhat across model conditions. More generally, we observed a range of RAWR γ settings that returned the highest PR-AUC, where the range typically spanned around one to two orders of magnitude.

Performance comparisons on long-gap-length model conditions. Similar performance outcomes were observed on the long-gap-length simulations, as compared to the medium-gap-length simulations in the rest of our simulation study. RAWR returned PR-AUC that was comparable or better than bootstrap with improvements amounting to as much as .236. RAWR's PR-AUC advantage was larger on model conditions with greater

Supplementary Table S3: **Simulation study: RAWR support estimation using alternative estimation/re-estimation methods.** We compared RAWR support estimation using two different estimation/re-estimation methods: either MAFFT and RAxML(MAFFT) or ClustalW and RAxML(ClustalW). For each of the two methods, aggregate PR-AUC is shown across all replicate datasets of each model condition ($n = 20$).

**Model condition**	**RAWR using**	
	**MAFFT and RAxML(MAFFT)**	**ClustalW and RAxML(ClustalW)**
10.A	0.996	0.997
10.B	0.990	0.991
10.C	0.977	0.988
10.D	0.968	0.992
10.E	0.925	0.964

Supplementary Table S4: **Simulation study: RAWR support estimation using different choices for reversal probability γ.** Aggregate PR-AUC is reported across all replicate datasets of each 10-taxon model condition ($n = 20$). PR-AUC values within 0.015 of the maximum reported in a model condition are shown in bold.

**Model condition**	**Reversal probability γ**						
	1×10^{-3}	1×10^{-2}	2×10^{-2}	5×10^{-2}	1×10^{-1}	2×10^{-1}	3×10^{-1}
10.A	**0.997**	**0.998**	**0.998**	**0.996**	**0.994**	**0.986**	0.980
10.B	**0.994**	**0.990**	**0.991**	**0.990**	**0.987**	**0.985**	0.977
10.C	0.942	0.941	0.950	**0.977**	**0.968**	0.957	0.901
10.D	**0.977**	**0.982**	**0.978**	0.95	0.944	0.935	0.934
10.E	**0.969**	**0.978**	**0.971**	**0.983**	0.929	0.923	0.922

Supplementary Table S5: **Simulation study: PR-AUC comparison of bootstrap and RAWR methods on 10-taxon long-gap-length model conditions.** MAFFT and RAxML(MAFFT) were used to perform MSA and tree estimation/re-estimation, respectively. Each method's PR-AUC is reported as an aggregate across all replicate datasets for a model condition ($n = 20$). PR-AUC values within 0.01 of the maximum observed in a model condition are highlighted in bold.

**Model Condition**	**PR-AUC**	
	**Bootstrap**	**RAWR**
10.long.A	**0.997**	**0.997**
10.long.B	**0.992**	**0.994**
10.long.C	0.904	**0.937**
10.long.D	0.829	**0.949**
10.long.E	0.552	**0.788**

evolutionary divergence. Both methods returned somewhat lower PR-AUC on the long-gap-length model conditions as compared to the medium-gap-length model conditions, but RAWR's PR-AUC improvements over bootstrap were largely unaffected by the gap length distribution used for simulation. This suggests that RAWR's performance is robust to increasing dependence between neighboring sites due to longer insertions and deletion events.

PR-AUC comparisons of aLRT and RAWR methods on simulated and empirical benchmarking datasets. RAWR consistently returned PR-AUC improvements relative to both aLRT methods across all of empirical benchmarking datasets and all model conditions in our simulation study (Supplementary Tables S6 and S7). Furthermore, RAWR's PR-AUC advantage tended to increase as model conditions grew in evolutionary divergence.

The original aLRT method was proposed to address the computational overhead of the standard phylogenetic bootstrap method, which requires re-estimation across multiple (and often many) bootstrap replicate datasets. The computational efficiency of the aLRT method is obtained using statistical approximations that represent a potential tradeoff in terms of type I/II error. Our findings support these observations, as the PR-AUC returned by both aLRT methods also underperformed traditional bootstrap support estimation (cf. Tables 3 and 4 in the main manuscript). We note one critical difference between our study and the study of Anisimova and Gascuel [1]: the former provided estimated annotation MSAs as input to phylogenetic support estimation methods under study , whereas the latter utilized true alignments. The relative performance comparisons of aLRT and RAWR can be attributed in part to the major impact of MSA quality on downstream phylogenetic and phylogenetic support estimation.

PR-AUC comparisons of TBE and RAWR methods on simulated and empirical benchmarking datasets. Based on PR-AUC, RAWR consistently outperformed the original TBE method [7] across all empirical benchmarks and all model conditions in our simulation study, with the exception of the least divergent 100-taxon model condition where

Supplementary Table S6: **Simulation study: PR-AUC comparison of aLRT and RAWR methods for phylogenetic support estimation.** We used PhyML [2] to run two types of aLRT analyses: (1) support estimation for a "free" tree topology that was also estimated as part of the analysis, and (2) support estimation for a RAxML-inferred tree topology. The latter methodology for obtaining an annotation tree is identical to the approach used in all other experiments in our study, and its PR-AUC performance is therefore directly comparable to other simulation study experiments. PR-AUC values within 0.01 of the maximum observed in a model condition are highlighted in bold. Table layout and description are otherwise identical to Table S5.

	**PR-AUC**		
**Model Condition**	**aLRT with free topology**	**aLRT with fixed topology**	**RAWR**
10.A	0.952	0.939	**0.996**
10.B	0.884	0.884	**0.990**
10.C	0.722	0.745	**0.977**
10.D	0.757	0.784	**0.968**
10.E	0.631	0.621	**0.925**
50.A	0.979	0.980	**0.997**
50.B	0.960	0.961	**0.994**
50.C	0.870	0.876	**0.989**
50.D	0.711	0.710	**0.988**
50.E	0.548	0.556	**0.997**
100.A	**0.986**	**0.987**	**0.993**
100.B	0.976	0.969	**0.991**
100.C	0.775	0.773	**0.982**
100.D	0.663	0.670	**0.983**
100.E	0.592	0.593	**0.986**

Supplementary Table S7: **Empirical study: PR-AUC comparison of aLRT and RAWR methods on CRW benchmarking datasets.** PR-AUC values within 0.01 of the maximum observed in a model condition are highlighted in bold. Table layout and description are otherwise identical to Table S6.

	**PR-AUC**		
**Dataset**	**aLRT (free topology)**	**aLRT (fixed topology)**	**RAWR**
IGIA	0.6696	0.7094	**0.7845**
IGIB	0.4904	0.5515	**0.8332**
IGIC2	0.6368	0.7242	**0.8808**
IGID	0.7998	0.7299	**0.8524**
IGIE	0.6832	0.6864	**0.8206**
IGIIA	0.7774	0.8036	**0.9053**

both methods returned comparable PR-AUC (Supplementary Tables S8 and S9).

TBE support calculation is downstream of input data resampling. The original TBE support estimation method utilized standard bootstrap resampling for the latter (which we refer to as "TBE+bootstrap"), but, as noted by Lemoine *et al.* [7], the orthogonality of these two problems allows other resampling techniques to be readily substituted. We therefore included a third method that paired TBE support calculation with RAWR resampling, which we refer to as "TBE+RAWR". TBE+RAWR returned comparable or improved PR-AUC compared to TBE+bootstrap, with the greatest improvements on the most divergent model conditions.

However, neither TBE method outperformed standard RAWR support estimation, which uses a traditional binary test for bipartition presence/absence to calculate phylogenetic support (like the phylogenetic bootstrap support method). We also did not observe PR-AUC comparisons that suggested a type I/II error advantage for the original TBE method (i.e., TBE+bootstrap) over traditional phylogenetic bootstrap support estimation (cf. Tables 3 and 4). Our findings differ from the study of Lemoine *et al.* [7], which we attribute to the following factors. As noted above, a major difference between the two studies is MSA quality: the former utilizes estimated MSAs and the latter utilizes true MSAs (modulo a noise injection model that shuffles nucleotide/amino acid homologies within true MSA columns but preserves true nucleotide/amino acid homologies). Furthermore, [7] noted that, by definition of the bipartition transfer distance, TBE support is always greater than or equal to traditional bootstrap support for a given set of inputs. Based on our findings, we conjecture that an optimistic support measure is beneficial for addressing type II error (i.e., low or no support for a true bipartition) but could be counterproductive for type I error (i.e., high support for a false bipartition). Estimated MSA accuracy is known to impact both in the context of phylogenetic estimation [11, 10, 12]. Ideally, TBE's innovative use of the bipartition transfer distance would be counterbalanced by "inverse" edit operation(s) that "undo" presence assessments for incorrect bipartitions (i.e., pessimistic adjustment of support to help address type I error).

Supplementary Table S8: **Simulation study: PR-AUC comparison of TBE with bootstrap resampling, TBE with RAWR resampling, and RAWR.** TBE was used to estimate phylogenetic support using two different resampling approaches: either (1) standard bootstrap resampling, which corresponds to the method originally proposed and studied by Lemoine *et al.* [7], or (2) RAWR resampling. The former is denoted "TBE with bootstrap resampling", and the latter is denoted "TBE with RAWR resampling". For comparison purposes, RAWR resampling and re-estimation was also run as a third method (denoted "RAWR"), and we used the same methodology as elsewhere in our study (i.e., using a standard branch presence/absence calculation to assess phylogenetic support).

	**PR-AUC**		
**Model Condition**	**TBE with bootstrap resampling**	**TBE with RAWR resampling**	**RAWR**
10.A	0.943	0.982	**0.996**
10.B	0.913	0.959	**0.990**
10.C	0.773	0.894	**0.977**
10.D	0.823	0.924	**0.968**
10.E	0.670	0.869	**0.925**
50.A	0.986	0.983	**0.997**
50.B	0.965	0.967	**0.994**
50.C	0.888	0.943	**0.989**
50.D	0.785	0.950	**0.988**
50.E	0.655	0.968	**0.997**
100.A	**0.995**	**0.984**	**0.993**
100.B	**0.985**	0.962	**0.991**
100.C	0.806	0.922	**0.982**
100.D	0.722	0.934	**0.983**
100.E	0.680	0.930	**0.986**

Among other important assumptions (e.g., treating indels as missing data or an additional base/state and its implications concerning statistical consistency of traditional substitution model-based phylogenetic estimation/re-estimation [19]), theoretical guarantees about TBE and phylogenetic bootstrap support implicitly assume that input sequences are aligned without error. But incorrect sequence homology and other mis-alignments – commonplace in all real-world settings – will require a different set of theoretical and applied considerations. Our experiments suggest that sequence-aware resampling and re-estimation has an important role to play in phylogenetic support estimation.

Simulation study experiments with alternative random walk resampling procedure. The PR-AUC values returned by RAWR+teleport on the 10-taxon model conditions are shown in Table S10. RAWR+teleport had comparable performance to the standard RAWR method in terms of PR-AUC (cf. Table 3). For the model conditions in our experiment, downstream re-estimation and support calculations may be relatively tolerant of

Supplementary Table S9: **Empirical study: PR-AUC comparison of TBE and RAWR methods on CRW benchmarking datasets.** PR-AUC values within 0.01 of the maximum observed in a model condition are highlighted in bold. Table layout and description are otherwise identical to Table S6.

	PR-AUC		
**Dataset**	**TBE with bootstrap resampling**	**TBE with RAWR resampling**	**RAWR**
IGIA	0.7100	0.7195	**0.7845**
IGIB	0.6194	0.7232	**0.8332**
IGIC2	0.7508	0.8559	**0.8808**
IGID	0.5915	0.8044	**0.8524**
IGIE	0.7235	0.7622	**0.8206**
IGIIA	0.8252	0.8524	**0.9053**

Supplementary Table S10: **PR-AUC performance of RAWR+teleport on 10-taxon model conditions.**

**Model Condition**	**PR-AUC**
10.A	0.998
10.B	0.99
10.C	0.978
10.D	0.966
10.E	0.964

"discontinuities" introduced by teleportation, at least relative to related noise injected by random reversals in a standard RAWR resampled replicate (see Discussion). Further experimentation will help to clarify tradeoffs between the different resampling methods.

Performance comparison of RAWR versus GUIDANCE2. We also compared the performance of RAWR versus GUIDANCE2, a state-of-the-art purpose-built fully parametric method for placing confidence intervals on estimated multiple sequence alignments. The application of an MSA confidence assessment method like GUIDANCE2 to the downstream task of phylogenetic support estimation differs from its original intended purpose. However, we note that GUIDANCE2 incorporates standard bootstrap resampling as a first step, and subsequent steps focus on guide tree re-estimation and other re-estimation tasks as part of progressive MSA re-estimation. For this reason, GUIDANCE2 can be seen as an adaptation of the standard bootstrap to MSA and tree re-estimation.

For both methods, MAFFT and/or RAxML(MAFFT) were used to estimate/re-estimate MSAs and phylogenetic trees. Similar to elsewhere in our simulation study, type I and II error was assessed based on PR-AUC comparisons.

The performance comparison between RAWR and GUIDANCE2 was qualitatively similar to that of RAWR and bootstrap. RAWR returned comparable or better PR-AUC compared to GUIDANCE2 on the simulated datasets, and RAWR's PR-AUC advantage over GUIDANCE2 tended to grow as model conditions grew larger and/or more divergent (Table S11). The performance comparison of RAWR and GUIDANCE2 was similar on the empirical benchmarks. GUIDANCE2 was the slowest method overall – even moreso than RAWR – due to the complexity of its special-purpose MSA re-estimation approach, and both GUIDANCE2 and RAWR required more main memory compared to bootstrap (Supplementary Figure S1). As shown in Supplementary Table S12, RAWR also outperformed GUIDANCE2 on all empirical benchmarks except for IGIB. The average absolute difference of the two methods' PR-AUC values was 0.055. This discrepancy may be ascribed to the relative difficulty that the IGIB dataset presents: the worst PR-AUC values in our entire study were observed on this dataset. One primary factor for this outcome is the high gappiness of the reference alignments for the IGIB and IGIA datasets (i.e., the fraction of the reference alignment that consists of indels), as compared to every other dataset in our study. RAWR resampling of datasets with high gappiness may require additional safeguards to mitigate "de-synchronization" (see Discussion).

Finally, we note that GUIDANCE2 is purpose-built for MSA re-estimation, whereas bootstrap and RAWR are general purpose non-parametric resampling methods (inasmuch as both resample an MSA without utilizing an explicit parametric model). Despite this, RAWR was able to match or exceed GUIDANCE2's PR-AUC performance.

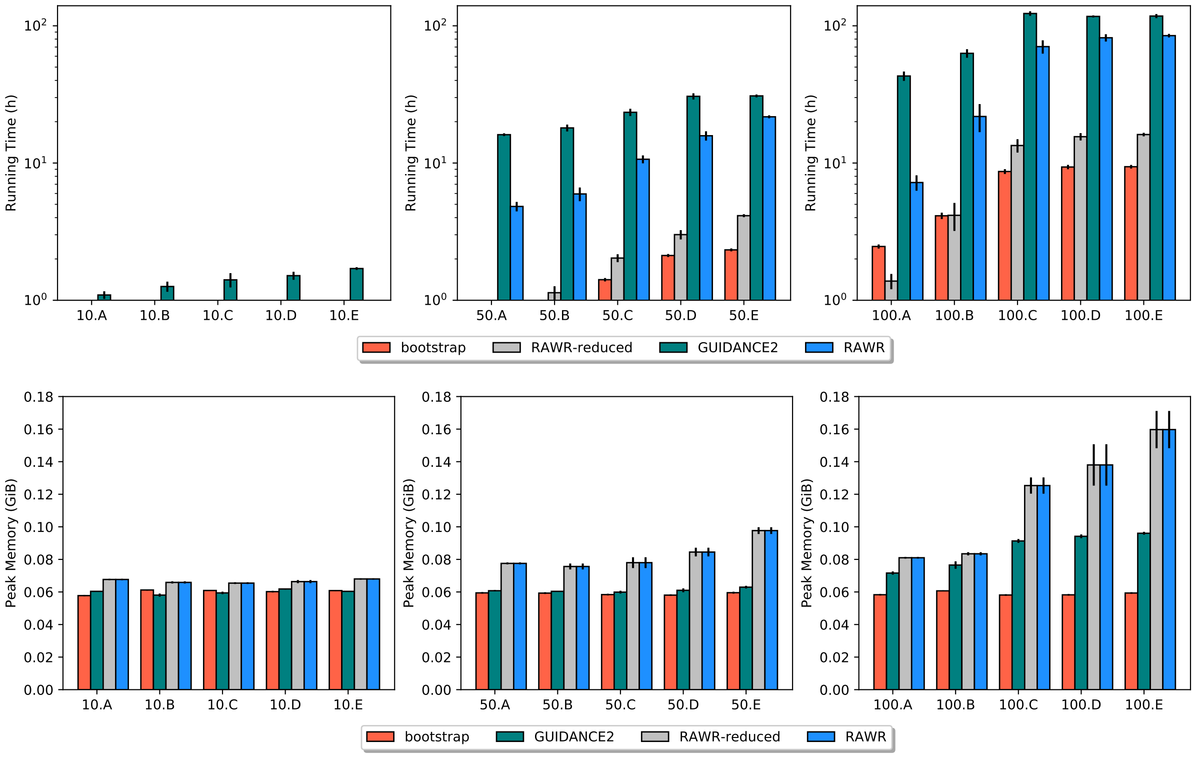

Supplementary Figure S1: **Simulation study: runtime and memory usage comparison of phylogenetic support estimation methods.** Results are shown for the 10-taxon, 50-taxon, and 100-taxon model conditions in the left, middle, and right columns, respectively. The 10-taxon model conditions are arranged on the x-axis from left to right in order of generally increasing evolutionary divergence; the 50-taxon and 100-taxon model conditions are similarly arranged. In each panel, average serial runtime or peak memory usage across all replicate datasets in the model condition is shown along with standard error bars ($n = 20$). In the top row of panels, the y-axis shows serial runtime in hours and is in log-scale. In the bottom row of panels, the y-axis shows peak memory usage in GiB and is in absolute scale.

Supplementary Table S11: **Simulation study: PR-AUC comparison of GUIDANCE2 and RAWR phylogenetic support estimation methods.** MAFFT and RAxML(MAFFT) were used to perform MSA and tree estimation/re-estimation, respectively. We report each method's aggregate PR-AUC across all replicate datasets for a model condition ($n = 20$). For each model condition, the top PR-AUC values within an absolute difference of 0.01 are shown in bold.

**Model**	**PR-AUC**	
**condition**	**GUIDANCE2**	**RAWR**
10.A	**0.989**	**0.996**
10.B	**0.983**	**0.990**
10.C	0.921	**0.977**
10.D	0.939	**0.968**
10.E	**0.997**	**0.997**
50.B	**0.994**	**0.994**
50.C	0.975	**0.989**
50.D	0.942	**0.988**
50.E	0.837	**0.997**
100.A	**0.988**	**0.993**
100.B	**0.993**	**0.991**
100.C	0.939	**0.982**
100.D	0.894	**0.983**
100.E	0.881	**0.986**

Supplementary Table S12: **Empirical study: PR-AUC comparison of GUIDANCE2 and RAWR methods for phylogenetic support estimation.** MAFFT and RAxML(MAFFT) were used to perform MSA and tree estimation/re-estimation, respectively. For each empirical dataset, the top PR-AUC value is shown in bold.

	**PR-AUC**	
**Dataset**	**GUIDANCE2**	**RAWR**
IGIA	0.705	**0.804**
IGIB	**0.737**	0.695
IGIC2	0.874	**0.957**
IGID	0.740	**0.884**
IGIE	0.777	**0.808**
IGIIA	0.870	**0.884**

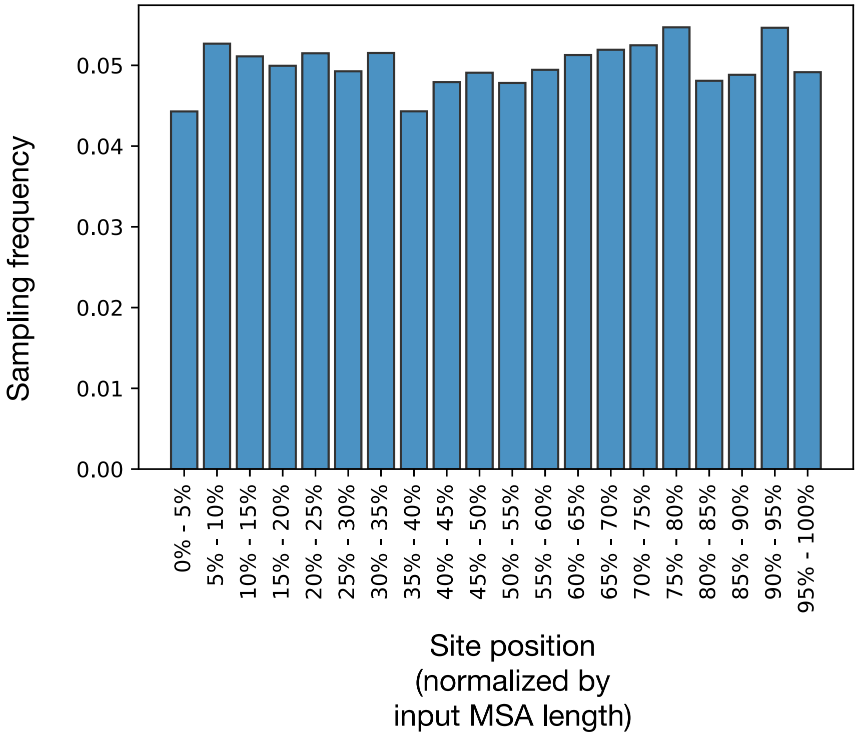

Supplementary Figure S2: **Frequency histogram of sites sampled by RAWR resampling procedure.** Results are shown for the 10.E model condition, where the histogram aggregates results across all model condition replicates ($n = 20$). Absolute site position is normalized by input MSA length to obtain a relative site position, and sampling frequency is reported for all sites having relative position within 5%-width intervals.

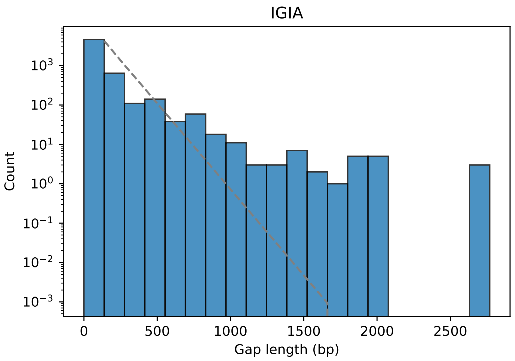

(a) IGIA

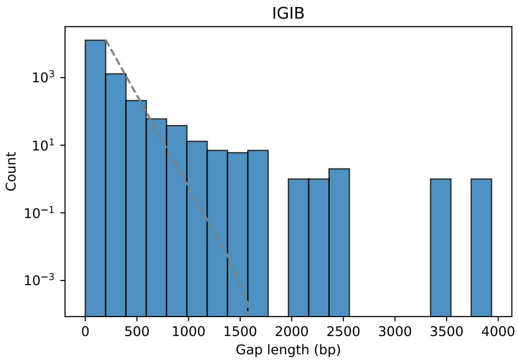

(b) IGIB

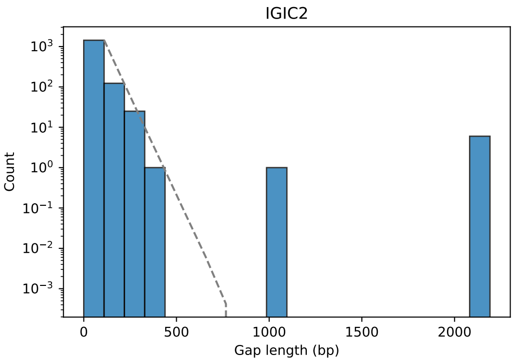

(c) IGIC2

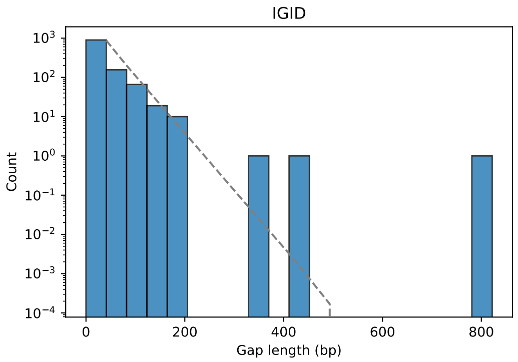

(d) IGID

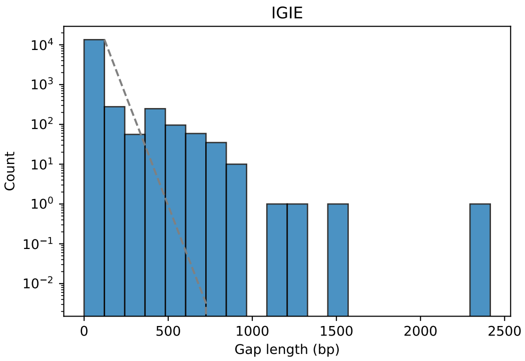

(e) IGIE

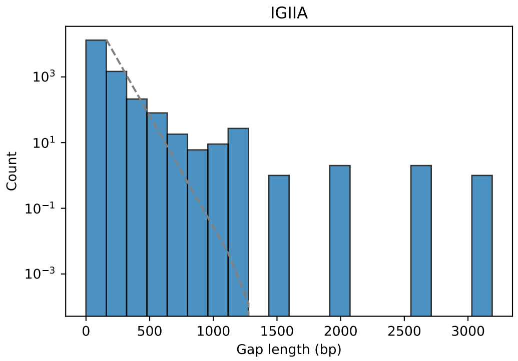

(f) IGIIA

Supplementary Figure S3: **Gap length histograms for the CRW datasets' reference MSAs.** Histogram counts are shown in log-scale. The reference alignments were manually curated and downloaded from the Comparative RNA Website (CRW) database (see Methods section for details). We used version 3.6.2 of the R stats package and the R version 4.0.4 software to fit geometric distributions to the gap length histograms. Fitted geometric distributions are shown as dashed lines. The fitted geometric distribution parameter values for the IGIA, IGIB, IGIC2, IGID, IGIE, and IGIIA datasets are 0.010, 0.012, 0.022, 0.033, 0.025, and 0.015, respectively.

References

[1] Anisimova, M. and Gascuel, O. (2006). Approximate likelihood-ratio test for branches: a fast, accurate, and powerful alternative. *Systematic Biology*, **55**(4), 539–552.

[2] Guindon, S., Dufayard, J.-F., Lefort, V., Anisimova, M., Hordijk, W., and Gascuel, O. (2010). New algorithms and methods to estimate maximum-likelihood phylogenies: assessing the performance of PhyML 3.0. *Systematic Biology*, **59**(3), 307–321.

[3] Katoh, K. and Standley, D. M. (2013). MAFFT multiple sequence alignment software version 7: improvements in performance and usability. *Molecular Biology and Evolution*, **30**(4), 772–780.

[4] Kozlov, A. M., Aberer, A. J., and Stamatakis, A. (2015). ExaML version 3: a tool for phylogenomic analyses on supercomputers. *Bioinformatics*, **31**(15), 2577–2579.

[5] Lamichhaney, S., Berglund, J., Almén, M. S., Maqbool, K., Grabherr, M., Martinez-Barrio, A., Promerová, M., Rubin, C.-J., Wang, C., Zamani, N., Grant, B. R., Grant, P. R., Webster, M. T., and Andersson, L. (2015). Evolution of Darwin's finches and their beaks revealed by genome sequencing. *Nature*, **518**(7539), 371–375.

[6] Landan, G. and Graur, D. (2007). Heads or tails: a simple reliability check for multiple sequence alignments. *Molecular Biology and Evolution*, **24**(6), 1380–1383.

[7] Lemoine, F., Entfellner, J.-B. D., Wilkinson, E., Correia, D., Felipe, M. D., De Oliveira, T., and Gascuel, O. (2018). Renewing Felsenstein's phylogenetic bootstrap in the era of big data. *Nature*, **556**(7702), 452–456.

[8] Li, H. and Durbin, R. (2009). Fast and accurate short read alignment with Burrows-Wheeler transform. *Bioinformatics*, **25**(14), 1754–1760.

[9] Li, H., Handsaker, B., Wysoker, A., Fennell, T., Ruan, J., Homer, N., Marth, G., Abecasis, G., and Durbin, R. (2009). The sequence alignment/map format and SAMtools. *Bioinformatics*, **25**(16), 2078–2079.

[10] Liu, K., Raghavan, S., Nelesen, S., Linder, C. R., and Warnow, T. (2009). Rapid and accurate large-scale coestimation of sequence alignments and phylogenetic trees. *Science*, **324**(5934), 1561–1564.

[11] Liu, K., Warnow, T. J., Holder, M. T., Nelesen, S. M., Yu, J., Stamatakis, A. P., and Linder, C. R. (2012). SATé-II: Very fast and accurate simultaneous estimation of multiple sequence alignments and phylogenetic trees. *Systematic Biology*, **61**(1), 90–106.

[12] Nelesen, S. M., Liu, K., Zhao, D., Linder, C. R., and Warnow, T. (2008). The effect of the guide tree on multiple sequence alignments and subsequent phylogenetic analysis. In *Pacific Symposium on Biocomputing*, volume 13, pages 25–36.

[13] Rodriguez, F., Oliver, J., Marin, A., and Medina, J. (1990). The general stochastic model of nucleotide substitution. *Journal of Theoretical Biology*, **142**, 485–501.

[14] Sanderson, M. J. (2003). r8s: inferring absolute rates of molecular evolution and divergence times in the absence of a molecular clock. *Bioinformatics*, **19**(2), 301–302.

[15] Scheet, P. and Stephens, M. (2006). A fast and flexible statistical model for large-scale population genotype data: Applications to inferring missing genotypes and haplotypic phase. *The American Journal of Human Genetics*, **78**(4), 629 – 644.

[16] Sela, I., Ashkenazy, H., Katoh, K., and Pupko, T. (2015). GUIDANCE2: accurate detection of unreliable alignment regions accounting for the uncertainty of multiple parameters. *Nucleic Acids Research*, **43**(W1), W7–W14.

[17] Stamatakis, A. (2014). RAxML version 8: a tool for phylogenetic analysis and post-analysis of large phylogenies. *Bioinformatics*, **30**(9), 1312–1313.

[18] Wang, W., Smith, J., Hejase, H. A., and Liu, K. J. (2018). Non-parametric and semi-parametric support estimation using sequential resampling random walks on biomolecular sequences. In *RECOMB International conference on Comparative Genomics*, pages 294–308. Springer.

[19] Warnow, T. (2012). Standard maximum likelihood analyses of alignments with gaps can be statistically inconsistent. *PLoS Currents*, **4**.
